# Supplementary material for: LAT condensation gates PLCγ1 activation via bimodal LAT phosphorylation
Source: Proc Natl Acad Sci U S A. Author manuscript; Available in PMC 2026 Oct 26. (PMC13643308; doi:10.1073/pnas.2614738123)
Supplement: Supplementary file 1 [file NIHMS2214831-supplement-Supplementary_Material.pdf]

Supporting Information for

LAT condensation gates PLCγ1 activation via bimodal LAT phosphorylation

He Ren[1,5], Hyun-Ro Lee[1,5], Yannick A.D. Omar[2], Joseph B. DeGrandchamp[1], Chien-Lun Hung[3], Timothy J. Eisen[1,3], Dimitrios Stamou[4], John Kuriyan[3], Arup K. Chakraborty[2], Jay T. Groves[1,*]

[1] Department of Chemistry, University of California, Berkeley, CA, USA, 94720
[2] Department of Chemical Engineering, Massachusetts Institute of Technology, Cambridge, MA, USA, 02139
[3]Department of Biochemistry, Vanderbilt University School of Medicine, Nashville, TN, USA, 37240
[4] Department of Chemistry, University of Copenhagen, Copenhagen, Denmark, DK-2100
[5] These authors contributed equally
*Corresponding author: Jay T. Groves
Email: jtgroves@lbl.gov

This PDF file includes:

Supporting text
Figures S1 to S8
SI References

Supporting Information Text

Materials and Methods

<u>Protein expression and purification</u>

PLCγ1 Mus musculus full-length PLCγ1 with C-terminal mNeonGreen tag was transiently expressed in HEK293 cells using Lipofectamine 3000 (Invitrogen) following the manufacturer's protocol. The clarified lysate was collected and directly used in the experiment following previous protocol (1). The exact concentration of PLCγ1-mNeonGreen was calibrated using purified mNeonGreen proteins (Figure S1A).

pLAT Human LAT cytosolic domain (27-233) with an N-terminal 10-Histidine tag was expressed in E. coli BL21 cells and purified using a Talon column, Mono Q, and Superdex 75 SEC column following previous protocol (1). Prephosphorylated LAT (pLAT) was obtained by incubating LAT with AncSZ kinase domain at a 1:160 molar ratio with 15 mM ATP and 20 mM MgCl2 at room temperature overnight. A Talon column followed by SEC was used to separate AncSZ.

BTK Human full-length BTK with an N-terminal 6-Histidine-SUMO tag was coexpressed with YopH, GroEL/ES, and trigger factor in E. coli BL21 cells (2). Following a Talon column, the chaperonins were dissociated in chaperonin dissociation buffer (20 mM HEPES, pH 7.0, 10 mM MgCl2, 5 mM ATP, and 150 mM KCl) at 4 °C for 1 h. After washing, His-SUMO-BTK was further purified with Superdex 75 SEC column. The His-SUMO tag of BTK was removed by incubation with His-SUMO protease (Sigma-Aldrich) at 200:1 molar ratio overnight at 4 °C. The cleaved His tag and His-SUMO protease were removed by a Talon column, and BTK was collected in the flow-through. BTK was labeled with Alexa Fluor 555 C2 Maleimide (Invitrogen) at 1:6 molar ratio overnight at 4 °C following the manufacturer's protocol. PD10 column followed by Superdex 200 SEC columns was used to remove excess dye molecules.

DAG sensor DAG-binding C1 domain of PKCθ with an N-terminal 6-Histidine-TEV site and C-terminal SNAP tag was expressed in E. coli BL21 cells and purified using a Talon column following previous protocol (1). His6-PKC-C1-SNAP was incubated with His-TEV protease (QB3 Macrolab) at 30:1 molar ratio overnight at 4 °C. The cleaved His tag and His-TEV protease were removed by a Talon column. PKC-C1-SNAP was labeled with SNAP-Surface Alexa Fluor 647 (New England Biolabs) at 1:2 molar ratio for 2 h at room temperature following the manufacturer's protocol. PD10 column followed by Superdex 75 SEC columns was used to remove excess dye molecules.

AncSZ The kinase domain of an ancestrally reconstructed Syk family kinase with an N-terminal 6-Histidine-PreScission Protease tag was coexpressed with YopH in E. coli BL21 cells and purified using a Talon column, Mono Q, and Superdex 75 SEC column (3). The

His tag of AncSZ was removed by incubation with an N-Terminal His tagged HRV-3C protease (Sigma-Aldrich) at 500:1 molar ratio at 4 °C for 24 h. The cleaved His tag and His-HRV-3C was removed by Talon column, and AncSZ was collected in the flow-through.

YopH Full-length Yersinia protein tyrosine phosphatase was expressed in E. coli BL21 cells and purified without affinity tag using CM Sepharose column and Superdex 75 SEC column following established protocol (4, 5).

Grb2 Human full-length Grb2 with an N-terminal 6-Histidine tag and a TEV site was expressed in E. coli BL21 cells and purified as described in (6).

SOS_PR The proline-rich domain of human SOS with an N-terminal 6-Histidine-MBP tag was expressed in E. coli BL21 cells and purified as described in (7).

<u>Functionalized supported membranes</u>

SUV Small unilamellar vesicles were made using 1,2-dioleoyl-sn-glycero-3-phosphocholine (DOPC), L-α-phosphatidylinositol-4,5-bisphosphate (Brain, Porcine) (ammonium salt) (PIP2), and 1,2-dioleoyl-sn-glycero-3-[(N-(5-amino-1-carboxypentyl)iminodiacetic acid)succinyl] (nickel salt) (Ni-NTA) from Avanti Polar Lipids. D-myo-Phosphatidylinositol 3,4,5-trisphosphate diC16 (ammonium salt) (PIP3) from Echelon Biosciences Inc. Lipids were mixed in an etched and dried round-bottom flask to yield 1 mmol total lipid at 96:4 (mol%) DOPC:Ni-NTA, 94:4:2 DOPC:Ni-NTA:PIP_2, 94:4:2 DOPC:Ni-NTA:PIP_3, or 90:4:2:4 DOPC:Ni-NTA:PIP_2:PIP_3. After mixing, lipids were dried by rotovap in a 37°C water bath for 2 min then at room temperature for 15 min. Later, the round-bottom flask was flushed with N_2 for 15 min. The lipid film was hydrated with 2 mL of milli-Q water for a final concentration of 0.5 mM total lipids. The lipid solution was added to a 5 mL polypropylene tube and treated by 3 mm stepped-tip sonication at 33% amplitude, 20 s sonication, 50 s rest, 5 cycles (Sonics VCX750) while submerged in ice. The lipid solution was clarified after sonication.

Reaction chamber Precision Schott D 263 M glass coverslips (#1.5H, 75 x 25 mm) were rinsed with milli-Q. Coverslips were then etched with piranha made with a 3:1 ratio of concentrated H_2SO_4 (Fisher Scientific) and 30% H_2O_2 (Fisher Scientific) for 5 min and rinsed extensively with milli-Q water. To make the experiment chamber, etched coverslips were blow-dried with N_2 and attached to 6-channel flow cells (sticky-Slide VI 0.4, ibidi).

SLB Supported lipid bilayers were made through SUV vesicle fusion. The 0.5 mM lipid solution was diluted to 0.25 mM in PBS (Fisher Scientific). Then, 150 µL of 0.25 mM lipid solution was added to each channel and incubated for 30 min at room temperature. After incubation, the channel was washed with 1 mL PBS and blocked with 1 mg/mL

blocker casein (Thermo Fisher Scientific) in PBS for 10 min. Each channel was washed with 1 mL PBS again.

LAT bilayer To attach LAT and phosphorylate LAT on the bilayer, 100 µL of 75 nM LAT or pre-phosphorylated LAT (pLAT) was added to each channel for 40 min. After washing the channel with 1 mL PBS, 100 µL of 1 mM ATP were added to the channel for 30 min to allow Ni-NTA and Histidine binding to reach a steady state (8). Due to the variations in SUV composition, including Ni-NTA lipid composition, LAT densities vary from day to day, and the variation is essentially unavoidable. Experiments conducted on the same day using the same batch of SUVs usually exhibit minimal variation in LAT density. Therefore, we only compare recruitment traces obtained on the same day.

Kinetic assay All kinetic measurements were performed in imaging buffer with 1X TBS, 2 mM MgCl2, 1 mM TCEP, 1 mM ATP, 1 mM sodium orthovanadate, 2 mM Trolox (UV treated), 10 mM BME, 0.1 mg/mL blocker casein. The channel was equilibrated with 200 nM of DAG sensor before injection of the reaction mixture. The default (1x) PLCγ1 concentration for all experiments was 15 nM, BTK was added at 50 nM, DAG sensor at 200 nM. To preincubate and preactivate His-BTK, 50 nM of His-BTK in 1X TBS, 2 mM MgCl2 was incubated for 40 min before the addition of the reaction mixture, after washing the channel with 1 mL PBS, 100 µL of 1 mM ATP were added to the channel for 30 min to allow Ni-NTA and Histidine binding to reach a steady state and His-BTK to autophosphorylate. Based on the kinetics measured by western blot (2), 30 min is sufficient for most BTK to be autophosphorylated.

<u>Microscope hardware and imaging acquisition</u>

TIRF imaging experiments were performed on an inverted Nikon Eclipse Ti microscope using a 100X Nikon oil-immersion TIRF objective (1.49 NA), and the 1.5X lens tube was also used in the experiment. 488/561/647 nm diode lasers (OBIS laser diode, Coherent) were controlled by a custom-built Solemere laser driver to excite fluorophores, and the images were acquired with an iXon Ultra 897 EMCCD camera (Andor Technology). All microscope hardware was controlled using Micro-Manager v4.0. Images were acquired with 15 s time lapse, 500 EM gain, at 100 ms exposure time. High laser intensity (20 mW out of the box) was used for single-molecule density experiments, and lower laser intensity (2 mW) was used for bulk density measurements. For all kinetic traces, the central 250*250 pixels were used to extract data and the mean intensity was analyzed in Fiji.

<u>Kinetic analysis</u>

Because SUV composition varies slightly from day to day, including the amounts of Ni-NTA lipids and phosphoinositides, LAT and BTK surface densities also vary between

experiments, and this variation is largely unavoidable. By contrast, experiments performed on the same day with the same SUV batch typically show minimal variation in LAT density. Therefore, we compare recruitment traces only within experiments conducted on the same day. Measurement errors were estimated from field-of-view segmentation as previously described (1). Briefly, each TIRF imaging field of view (2500 μm^2) was divided into 10 × 10 patches. The mean intensities of the central 5 × 5 patches were then used to calculate the mean and standard deviation of the kinetic traces. Additional independent experiments performed on other days are provided in Figure S4-S6 to illustrate experimental reproducibility.

<u>pLAT and PLCγ1 binding and unbinding model</u>

We analyzed the binding between phosphorylated LAT (pLAT) and PLCγ1 in Figure 2C using a simple reversible binding model. Since PLCγ1 predominantly binds to LAT pY132 by its nSH2 domain, we described the binding kinetics as

$$\frac{d[pLAT - PLC\gamma 1]_\sigma}{dt} = k_{on} \cdot ([pLAT]_{\sigma,total} - [pLAT - PLC\gamma 1]_\sigma) \cdot [PLC\gamma 1]_{sol} - k_{off} \cdot [pLAT - PLC\gamma 1]_\sigma$$

Because the total number of PLCγ1 molecules in solution was significantly higher than the number recruited to pLAT on the membrane, $[PLC\gamma 1]_{sol}$ was treated as a reservoir. Under this assumption, individual recruitment traces in Figure 2C were fitted to the following equation:

$$[pLAT - PLC\gamma 1]_\sigma(t) = [pLAT - PLC\gamma 1]_{\sigma,eq} \cdot (1 - e^{-k_{mem} \cdot t})$$

where

$$k_{mem} = k_{on} \cdot [PLC\gamma 1]_{sol} + k_{off}$$

From the values of k_{mem} obtained from the individual traces at different concentrations of $[PLC\gamma 1]_{sol}$, we determined $k_{on} = 5 \times 10^4\ \mathrm{M^{-1}s^{-1}}$ and $k_{off} = 1 \times 10^{-2}\ \mathrm{s^{-1}}$.

<u>PLCγ1 activation model</u>

To estimate the fraction of membrane-associated PLCγ1 molecules that have entered the active state (f_{act}), we used a minimal kinetic model describing PLCγ1 activation by BTK on the membrane as shown in Figure 3A. Concentration of autoinhibited PLCγ1 in solution is described as $[PLC]_{sol}$; density of membrane-bound and inactive PLCγ1 is described as $[PLC]_{\sigma,0}$; density of membrane-bound and active PLCγ1 is described as $[PLC]_{\sigma,act}$. Densities of membrane-associated PLCγ1 species are described by the following rate equations:

$$\frac{d[PLC]_{\sigma,0}}{dt} = k_{on} \cdot [PLC]_{sol} - (k_{off} + k_{act}) \cdot [PLC]_{\sigma,0}$$

$$\frac{d[PLC]_{\sigma,act}}{dt} = k_{act} \cdot [PLC]_{\sigma,0} - k_{off} \cdot [PLC]_{\sigma,act}$$

At steady state

$$\frac{[PLC]_{\sigma,act}}{[PLC]_{\sigma,0}} = \frac{k_{act}}{k_{off}}$$

The fraction of activated PLCγ1 on the membrane is therefore

$$f_{act} = \frac{[PLC]_{\sigma,act}}{[PLC]_{\sigma,total}} = \frac{[PLC]_{\sigma,act}}{[PLC]_{\sigma,0} + [PLC]_{\sigma,act}} = \frac{k_{act}}{k_{act} + k_{off}}$$

The timescale for reaching the steady-state density of activated PLCγ1 is determined by the characteristic time $\frac{1}{k_{act}+k_{off}}$. Assuming that the dissociation rate of inactive PLCγ1 is equal to that of activated PLCγ1, with $k_{off} = 0.01\ \mathrm{s}^{-1}$, we can estimate whether the system reaches steady state within the duration of the experiment.

For His-BTK, the density of activated BTK remains approximately constant throughout the reaction, allowing k_{act} to be treated as time-independent. Even for relatively small values of k_{act}, the system reaches 86.4% of its steady state activation around t = 200 s, at which point $k_{cat} \cdot f_{act}$ is effectively constant.

In contrast, for BTK recruited from solution, the density of activated BTK increases over time, leading to a time-dependent increase in k_{act}. This results in a gradual increase in f_{act}.

<u>Bimolecular reaction simplification from Michaelis–Menten mechanism</u>

As established from the previous section, $k_{cat} \cdot f_{act}$ is effectively constant for PLCγ1 activation by His-BTK after 200 s. To interpret kinetic measurements of PLCγ1 in the experiments described here, we consider a membrane surface Michaelis–Menten mechanism for PIP_2 hydrolysis to DAG:

$$\frac{d[DAG]_{\sigma}}{dt} = \frac{k_{cat} \cdot f_{act} \cdot [PLC]_{\sigma} \cdot [PIP_2]_{\sigma}}{K_M + [PIP_2]_{\sigma}}$$

Since we don't know the scale of K_M compared to $[PIP_2]_\sigma$, we can rearrange this equation and plot as

$$\frac{1}{[PLC]_\sigma} \cdot \frac{d[DAG]_\sigma}{dt} = \frac{k_{cat} \cdot f_{act} \cdot [PIP_2]_\sigma}{K_M + [PIP_2]_\sigma}$$

There are three possible cases, (1) $K_M \gg [PIP_2]_\sigma$, (2) $K_M \ll [PIP_2]_\sigma$,(3) K_M is similar to $[PIP_2]_\sigma$.

Case 1, if $K_M \gg [PIP_2]_\sigma$:

$$\frac{1}{[PLC]_\sigma} \cdot \frac{d[DAG]_\sigma}{dt} = \frac{k_{cat} \cdot f_{act} \cdot [PIP_2]_\sigma}{K_M + [PIP_2]_\sigma} \approx \frac{k_{cat} \cdot f_{act} \cdot [PIP_2]_\sigma}{\mathrm{K_M}}$$

We will observe that $\frac{1}{[PLC]_\sigma} \cdot \frac{d[DAG]_\sigma}{dt}$ increases linearly with $[PIP_2]_\sigma$.

Case 2, if $K_M \ll [PIP_2]_\sigma$:

$$\frac{1}{[PLC]_\sigma} \cdot \frac{d[DAG]_\sigma}{dt} = \frac{k_{cat} \cdot f_{act} \cdot [PIP_2]_\sigma}{K_M + [PIP_2]_\sigma} \approx k_{cat} \cdot f_{act}$$

We will observe that $\frac{1}{[PLC]_\sigma} \cdot \frac{d[DAG]_\sigma}{dt}$ stays constant with $[PIP_2]_\sigma$.

Case 3, if K_M is similar to $[PIP_2]_\sigma$, we will observe a non-linear relationship between $\frac{1}{[PLC]_\sigma} \cdot \frac{d[DAG]_\sigma}{dt}$ and $[PIP_2]_\sigma$.

As shown in Figure 3C, $\frac{1}{[PLC]_\sigma} \cdot \frac{d[DAG]_\sigma}{dt}$ increases linearly with $[PIP_2]_\sigma$, which supports that for active PLCγ1, $K_M \gg [PIP_2]_\sigma$. Thus, the catalytic reaction of PLCγ1 is limited by substrate binding, and we can simplify the kinetic description into the bimolecular reaction kinetics.

<u>Comparing rates of BTK and ZAP70 on LAT Y132 phosphorylation</u>

We measured the rate of BTK phosphorylating LAT Y132 (k_{BTK}) by using PLCγ1 recruitment as a sensor (Figure S8). BTK membrane density is calibrated in Figure S1C.

$$\frac{d[pLAT]_\sigma}{dt} = k_{BTK} \cdot [BTK]_\sigma \cdot [LAT]_\sigma$$

In our assay, BTK activation did not reach a steady state within the experimental timescale, so we used the highest mean measured rate $5 \cdot 10^{-5}\ \mu m^2 s^{-1}$ to approximate the fully activated, per-molecule catalytic rate of BTK.

To compare this with ZAP70, we referenced an earlier measurement of ZAP70 phosphorylating LAT, which yielded a rate of $10 \cdot 10^{-5}\ \mu m^2 s^{-1}$ while using Grb2 as the sensor. Importantly, the Grb2 sensor binds to fast-phosphorylating sites like Y171 and Y191 (9). Separate peptide assays have shown that the glycine residue preceding Y132 makes it ~10-fold slower for ZAP70 to phosphorylate than sites like Y171 and Y191 (10). Therefore, the estimated ZAP70 rate specifically for Y132 is approximately $1 \cdot 10^{-5}$ $\mu m^2 s^{-1}$.

This conclusion is further supported by an independent substrate selectivity study (11). In that "Kinase Library" screen (https://kinase-library.mit.edu/site), using the peptide sequence containing LAT Y132 (DDYHNPGY*LVVLPDS), BTK (71.79 percentile) was ranked as a more specific kinase for this substrate than ZAP70 (67.21 percentile). In contrast, ITK ranked lower (42.52 percentile).

<u>ITK positive feedback model simulation</u>

To simulate the model described in Figure 6, we first non-dimensionalize the governing equations such that $\alpha \to 1$ and $\mu_1 \to 1$. To obtain the steady-state solution, we use MATLAB's fsolve solver (MATLAB version: 25.1.0.2973910 (R2025a)). For the dynamical system, we integrate the equations forward in time using MATLAB's ode45 function until the non-dimensional time $t = 250$ is reached. To model the effect of ZAP70, we change the parameter β to a non-zero value at time $t = 50$. Similarly, we switch the parameter β back to zero at $t = 52$ to simulate the effect of a short duration of ZAP70 activity.

<u>Relationship to the HCK-based reconstitution system of DeGrandchamp et al.</u>

DeGrandchamp et al. (1) used a supported lipid bilayer assay containing membrane-tethered His-HCK to reconstitute LAT phosphorylation, PLCγ1 recruitment, and PLCγ1-mediated PIP_2 hydrolysis. Although His-HCK was primarily intended to phosphorylate LAT, it also phosphorylated PLCγ1 following its recruitment to the membrane. This approach established that PLCγ1 catalytic activity does not depend on the LAT condensation state; however, it did not allow for evaluation of the PLCγ1 activation (phosphorylation and autoinhibition release) process itself. In the present study, we addressed these questions by using prephosphorylated LAT and subsequently adding

BTK, a TEC family kinase closely related to ITK, thereby allowing the PLCγ1 activation process to be characterized in a near-native environment.

In the current reconstitution assay setup, both HCK and BTK are capable of phosphorylating LAT Y132 and PLCγ1 Y783. LAT Y132 phosphorylation was inferred from PLCγ1 membrane recruitment, whereas PLCγ1 Y783 phosphorylation was demonstrated by PLCγ1 catalytic activity. In the case of PLCγ2 activation in B cells, HCK recruitment by BTK has been identified as a tyrosine-kinase inhibitor resistance mechanism (12), providing another example of kinase cross-talk playing a functional role in PLCγ activation. The key feature in both B cells and our proposed mechanism for ITK in T cells is recruitment. Once recruited into the LAT condensate, kinase specificity is largely lost due to high local concentration (and activation). Subtle differences in kinase substrate specificity are not likely consequential once the kinases are localized in the condensate.

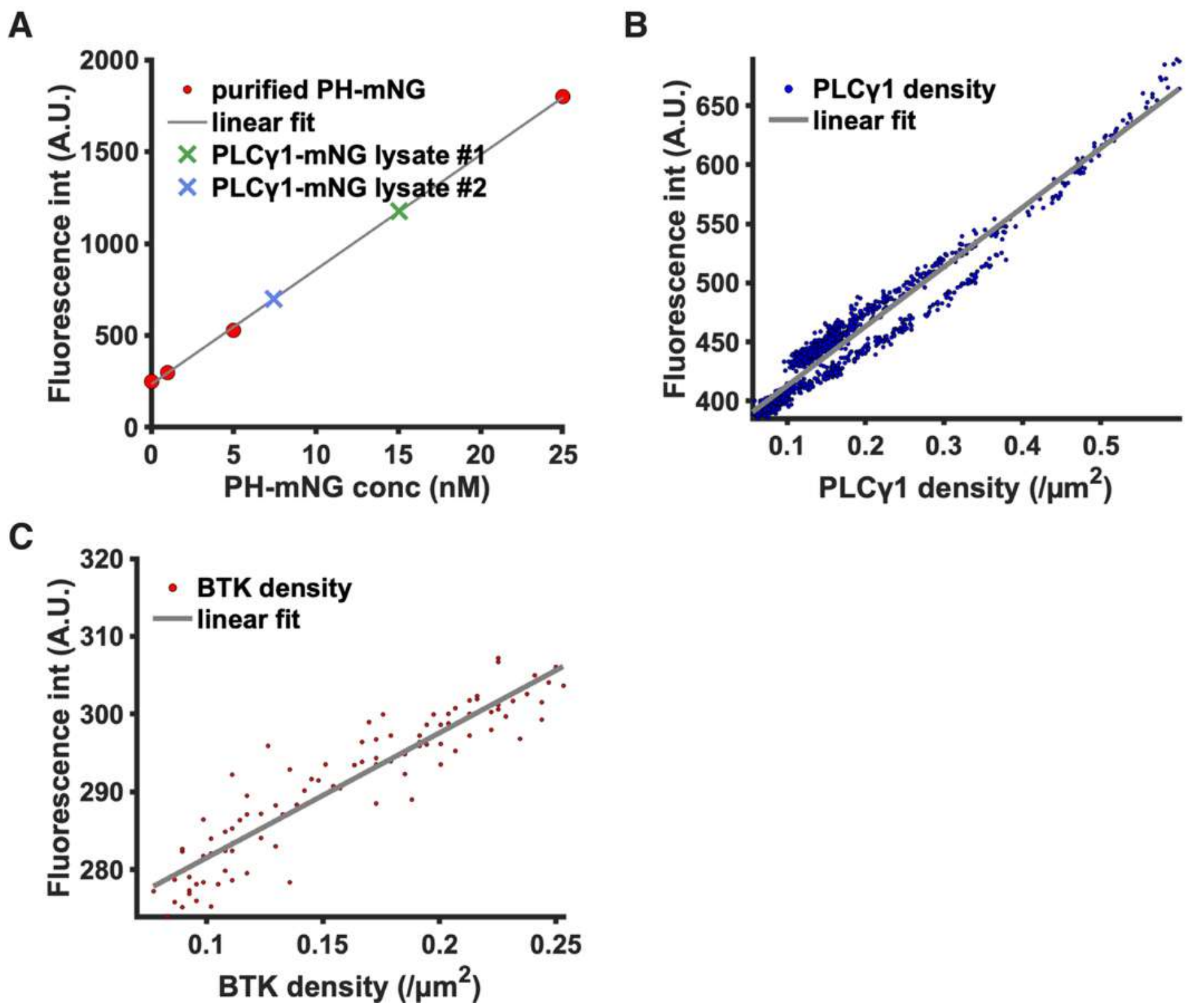

Figure S1. Protein concentration and membrane density calibration. (A) PLCγ1 concentration in lysate was calibrated against purified mNeonGreen-tagged protein using epifluorescence microscopy. (B) Calibration of PLCγ1 membrane density. (C) Calibration of BTK-555 membrane density.

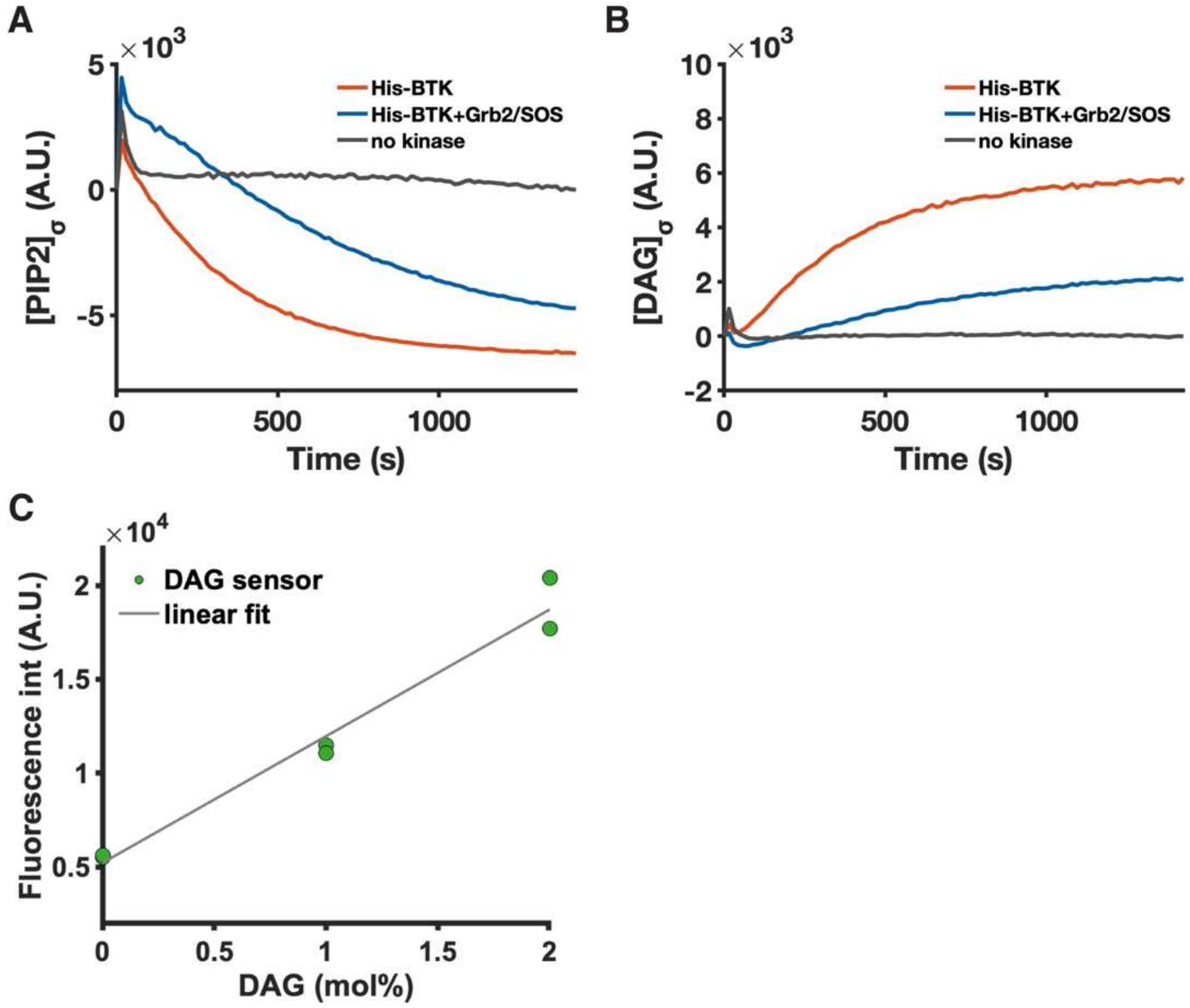

Figure S2. PIP_2 sensor and DAG sensor. (A-B) Example traces of the mean intensities of PIP_2 sensor and DAG sensor showing an inverse correlation. (C) Calibration of the DAG sensor ($R^2 = 0.97$).

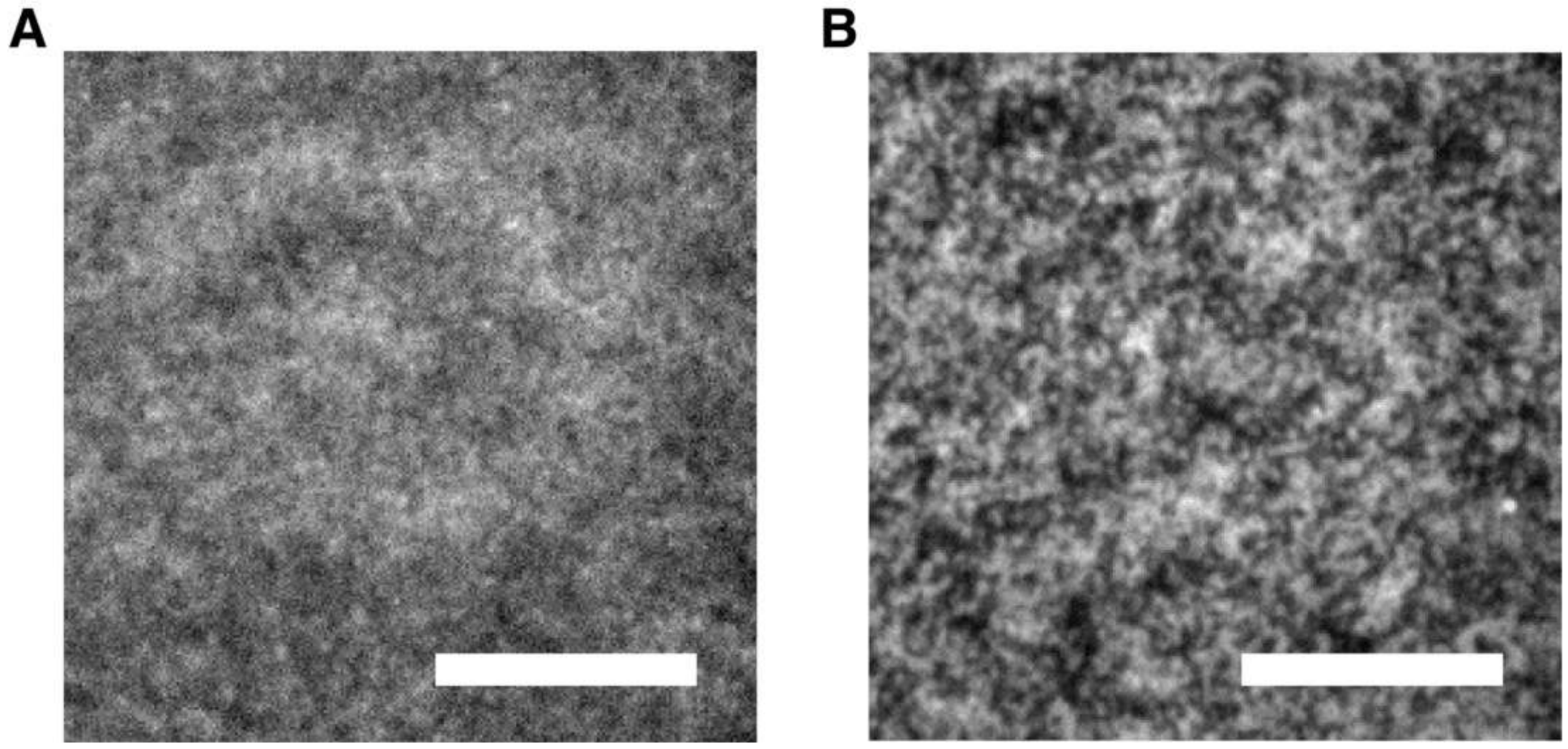

Figure S3. PLCγ1 in LAT condensate. Example images of PLCγ1 membrane recruitment without (A) and with (B) 5 μM Grb2 and 2 μM SOS_{PR}. (scale bars = 10 μm)

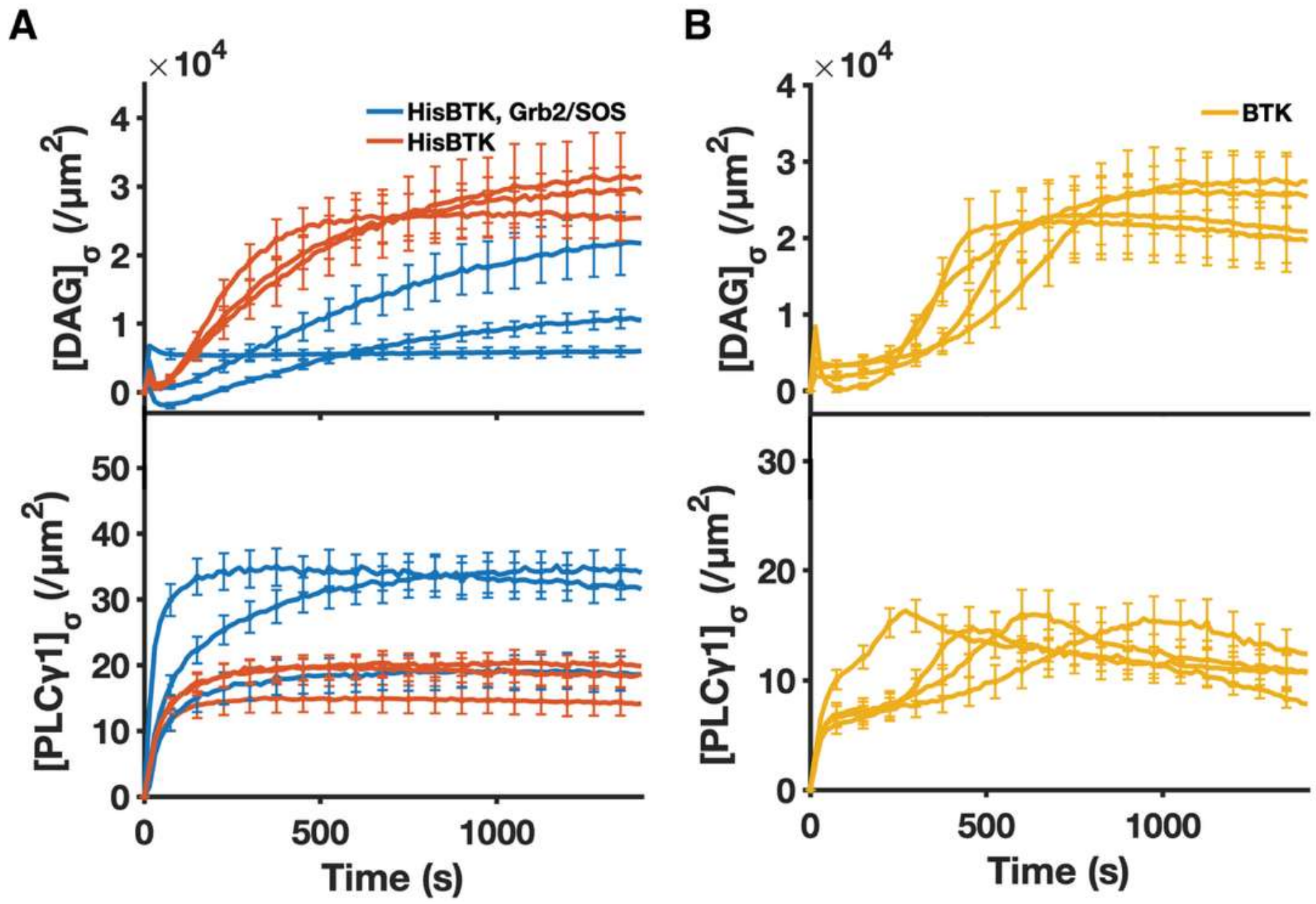

Figure S4. Repeats of kinetic measurements for (A) preincubated 50 nM His-BTK and (B) 50 nM wild-type BTK. (Membrane composition: 90:4:2:4 DOPC:Ni-NTA:PIP_2:PIP_3.)

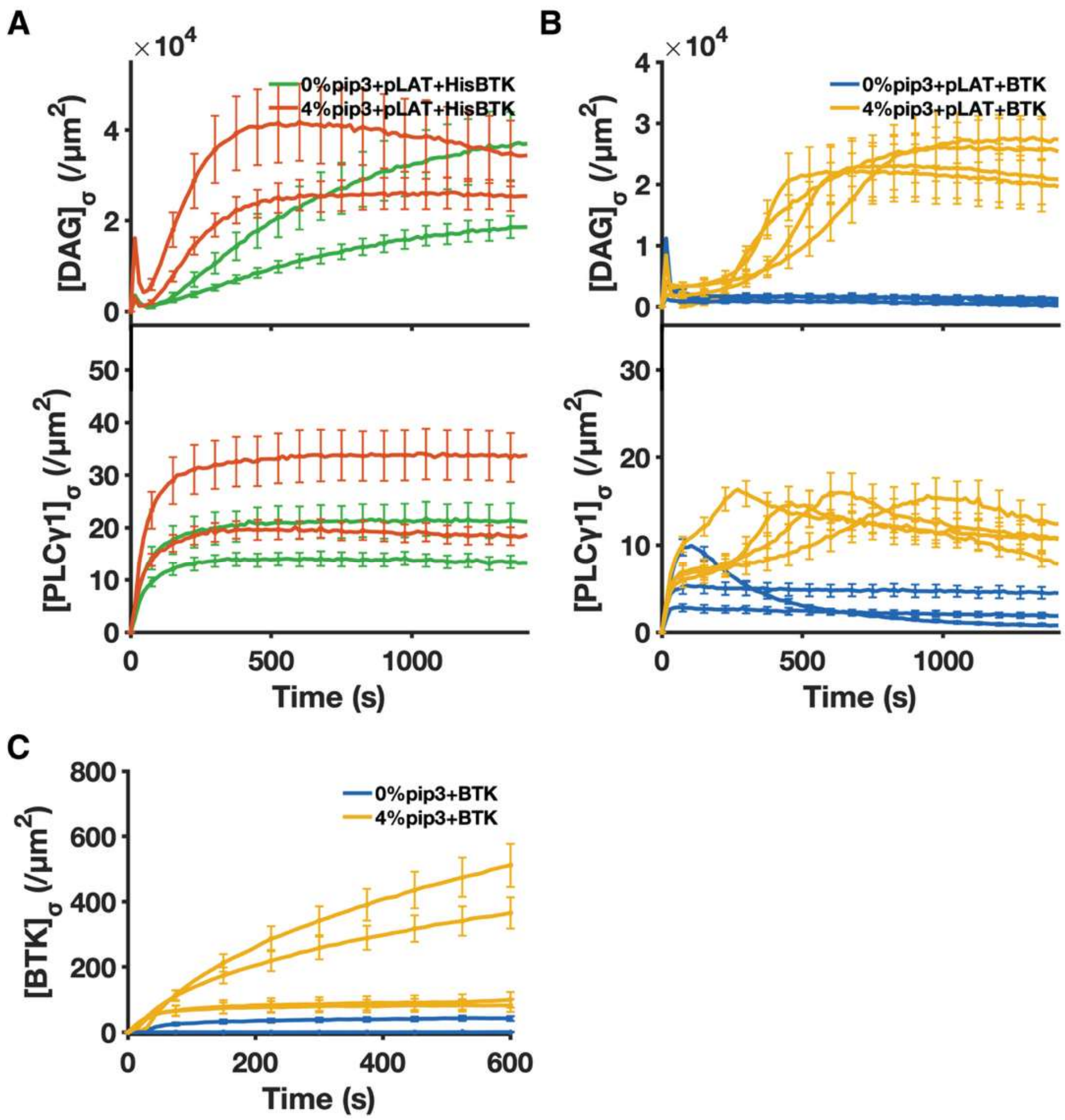

Figure S5. Dependence of His-BTK and wild-type BTK activation on PIP_3. (A) His-BTK activation is partially PIP_3-independent. DAG production in the absence of PIP_3 indicates that membrane tethering alone can activate His-BTK. The presence of PIP_3 did enhance the rate of PLCγ1 catalysis, suggesting either a higher fraction of activated PLCγ1 (f_{act}) or higher k_{cat}, possibly by better positioning of PLCγ1 on the membrane. (B) Wild-type BTK activation and membrane recruitment require PIP_3. PLCγ1-dependent DAG production indicates that BTK remained inactive without PIP_3. (C) Fluorescently labeled BTK exhibited significant membrane recruitment only in the presence of PIP_3. The variation in BTK membrane recruitment likely comes from PIP_3 variation in the SUV. (0% PIP_3: 94:4:2 DOPC:Ni-NTA:PIP_2; 4% PIP_3: 90:4:2:4 DOPC:Ni-NTA:PIP_2:PIP_3.)

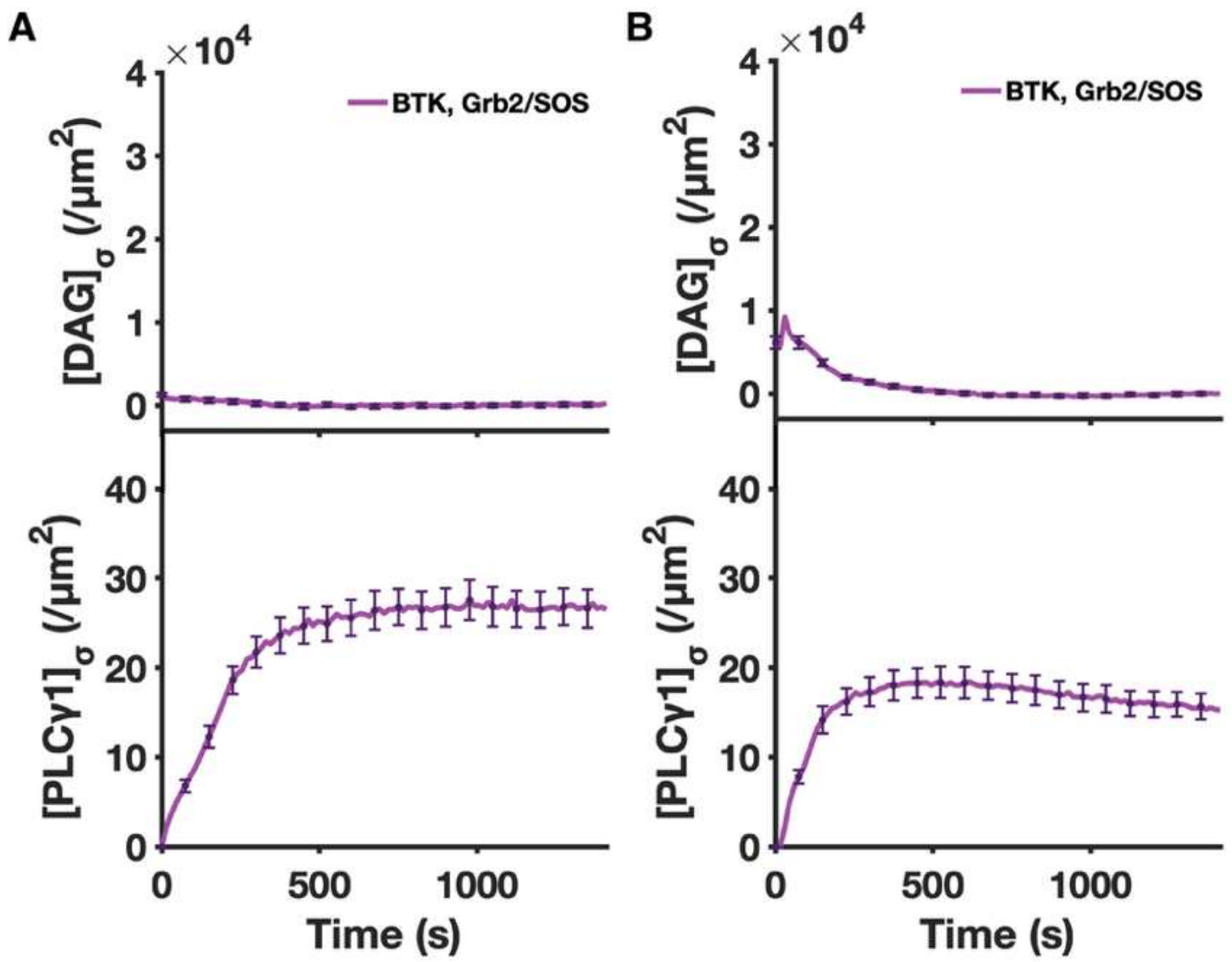

Figure S6. Two repeats of the wild-type BTK experiment with condensed LAT. pLAT was incubated at approximately 500 molecules/µm^2, PLCγ1 was added at 15 nM, and DAG sensor was added at 200 nM. BTK (50 nM), Grb2 (5 µM), and SOS_{PR} (2 µM) were added along with the reaction mixture. (Membrane composition: 90:4:2:4 DOPC:Ni-NTA:PIP_2:PIP_3.)

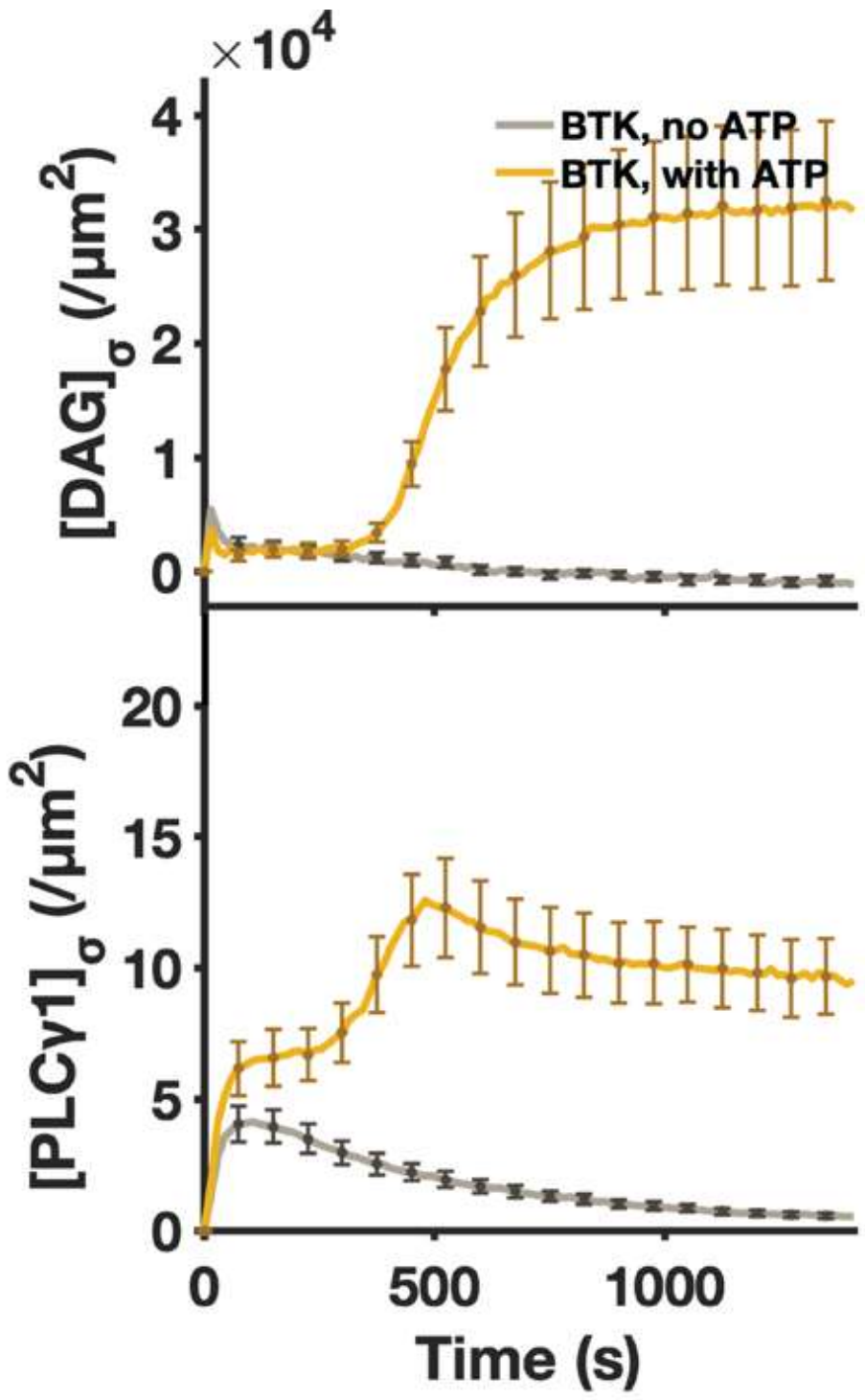

Figure S7. The yellow trace represents the standard imaging condition with ATP. The gray trace represents the experiment without ATP. pLAT was incubated at approximately 500 molecules/μm^2, PLCγ1 was added at 15 nM, and DAG sensor was added at 200 nM. 50 nM of BTK was added along with the reaction mixture. PLCγ1 recruitment in the no-ATP condition gradually decreased over time, likely because of dephosphorylation by trace phosphatase contamination in the kinase preparation. (Membrane: 90:4:2:4 DOPC:Ni-NTA:PIP_2:PIP_3.)

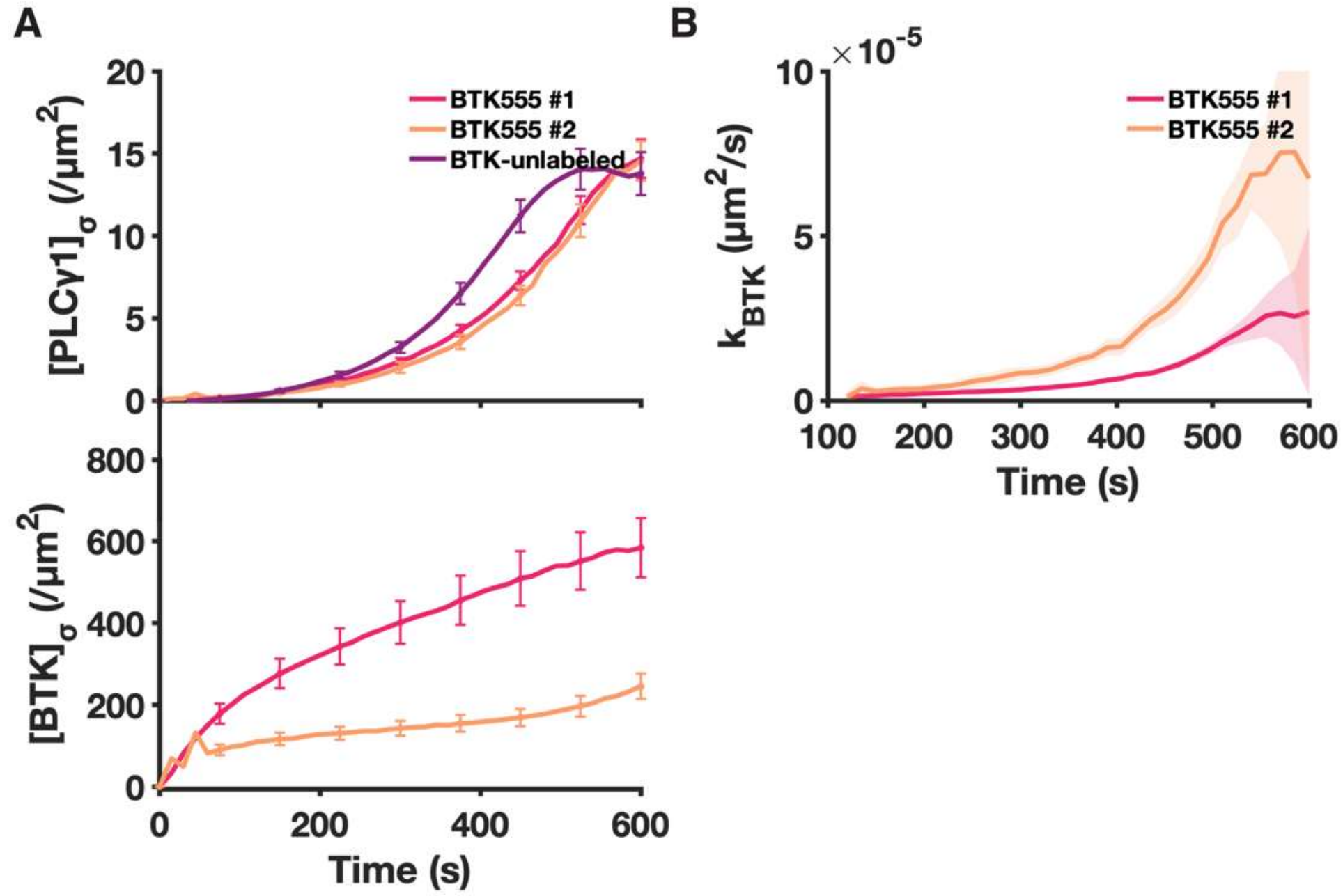

Figure S8. BTK phosphorylates LAT. (A) PLCγ1 membrane recruitment served as a readout for LAT Y132 phosphorylation. (B) Per-molecule catalytic analysis showed that BTK activation did not reach a steady state within the experimental timescale. Mean shown with shaded error. (Membrane composition: 90:4:2:4 DOPC:Ni-NTA:PIP_2:PIP_3.)

SI References

1. J. B. DeGrandchamp, S. V. Rajesh, L. J. N. Lew, J. T. Groves, Supported membrane assay probes PLCγ1 activity in LAT condensates. *bioRxiv* 10.64898/2026.01.23.701188, 2026.2001.2023.701188 (2026).
2. Q. Wang *et al.*, Autoinhibition of Bruton's tyrosine kinase (Btk) and activation by soluble inositol hexakisphosphate. *eLife* **4**, e06074 (2015).
3. H. T. Hobbs *et al.*, Saturation mutagenesis of a predicted ancestral Syk-family kinase. *Protein Science* **31**, e4411 (2022).
4. Z. Y. Zhang *et al.*, Expression, purification, and physicochemical characterization of a recombinant Yersinia protein tyrosine phosphatase. *Journal of Biological Chemistry* **267**, 23759–23766 (1992).
5. G. L. Waddell, E. E. Drew, H. P. Rupp, S. D. Hansen, Mechanisms controlling membrane recruitment and activation of the autoinhibited SHIP1 inositol 5-phosphatase. *Journal of Biological Chemistry* **299** (2023).
6. C.-W. Lin *et al.*, A two-component protein condensate of the EGFR cytoplasmic tail and Grb2 regulates Ras activation by SOS at the membrane. *Proceedings of the National Academy of Sciences* **119**, e2122531119 (2022).
7. W. Y. C. Huang *et al.*, Phosphotyrosine-mediated LAT assembly on membranes drives kinetic bifurcation in recruitment dynamics of the Ras activator SOS. *Proceedings of the National Academy of Sciences* **113**, 8218–8223 (2016).
8. J. A. Nye, J. T. Groves, Kinetic Control of Histidine-Tagged Protein Surface Density on Supported Lipid Bilayers. *Langmuir* **24**, 4145–4149 (2008).
9. W. Y. C. Huang, J. A. Ditlev, H.-K. Chiang, M. K. Rosen, J. T. Groves, Allosteric Modulation of Grb2 Recruitment to the Intrinsically Disordered Scaffold Protein, LAT, by Remote Site Phosphorylation. *Journal of the American Chemical Society* **139**, 18009–18015 (2017).
10. W.-L. Lo *et al.*, Slow phosphorylation of a tyrosine residue in LAT optimizes T cell ligand discrimination. *Nature Immunology* **20**, 1481–1493 (2019).
11. T. M. Yaron-Barir *et al.*, The intrinsic substrate specificity of the human tyrosine kinome. *Nature* **629**, 1174–1181 (2024).
12. K. Dhami *et al.*, Kinase-deficient BTK mutants confer ibrutinib resistance through activation of the kinase HCK. *Science Signaling* **15**, eabg5216.
